# Supplementary material for: Universality of Dicke superradiance in arrays of quantum emitters
Source: Nat Commun. 2022 Apr 27;13:2285. doi: 10.1038/s41467-022-29805-4 (PMC9046277; doi:10.1038/s41467-022-29805-4)
Supplement: Supplementary file 1 — Supplementary Information [file 41467_2022_29805_MOESM1_ESM.pdf]

Supplementary Information: Universality of Dicke superradiance in arrays of quantum emitters

Stuart J. Masson[1] and Ana Asenjo-Garcia[1]

[1]*Department of Physics, Columbia University, New York, NY 10027, USA*

This Supplementary Information contains information about the decay rates of 1D and 2D arrays as a function of interatomic distance, calculations regarding the role of Hamiltonian interactions and calculations regarding imperfections in the initial state, and in the array.

I. DECAY RATES AS A FUNCTION OF DISTANCE IN ORDERED ARRAYS

Generally, the variance of the eigenvalues $\{\Gamma_\nu\}$ increases with decreasing inter-atomic distances. However, this is not always strictly true. At some specific distances, there are geometric resonances that cause the decay rates to experience sudden changes [1–4], which leads to an increase in the variance, as shown in Supplementary Figure 1. These resonances are associated with far-field contributions to the interaction, and occur because certain decay channels become significantly brighter due to constructive interference. In 1D, the first revival occurs at $d = \lambda_0/2$. Extremely subradiant states do not exist for this distance, and thus this revival is not enough to enhance two-photon emission and superradiance. In 2D, for atoms polarized perpendicular to the surface, revivals occur at $d = \lambda_0/2$ and $d = \lambda_0/\sqrt{2}$. For these distances in 2D there are subradiant states. The revivals are strong enough to cause superradiance, leading to the non monotonic behavior of the critical distance with atom number observed in Fig. 4(b) in the main text. For atoms with polarization in the plane, far-field emission in the plane is forbidden in the direction that coincides with that of the polarization, greatly quenching the revivals.

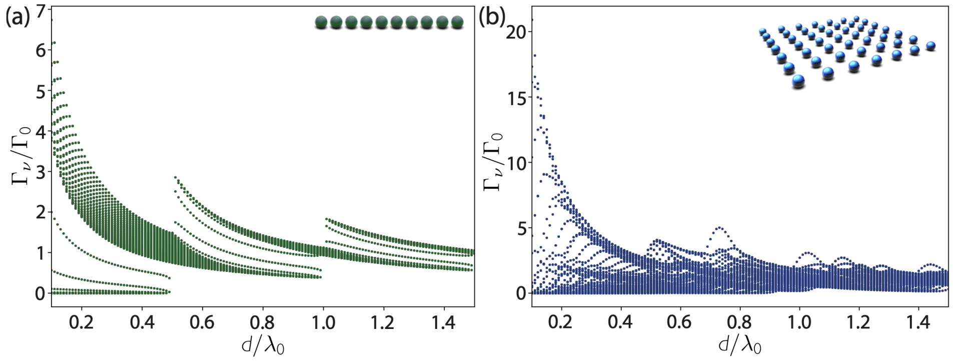

Supplementary Figure 1. Operator decay rates for 100 atoms arranged in a (a) chain and (b) 10×10 square array. Atoms are polarized (a) parallel to the array and (b) perpendicular to the array.

II. ROLE OF HAMILTONIAN INTERACTIONS IN DEPHASING

We consider the role of the Hamiltonian by considering a delay time between the first two photons and comparing to the case without a delay. We calculate

$$\frac{g^{(2)}(\tau)}{g^{(2)}(0)} = \frac{\sum\limits_{\nu,\mu=1}^{N} \Gamma_\nu \Gamma_\mu \left\langle \hat{\mathcal{O}}_\nu^\dagger e^{i\mathscr{H}\tau} \hat{\mathcal{O}}_\mu^\dagger \hat{\mathcal{O}}_\mu e^{-i\mathscr{H}\tau} \hat{\mathcal{O}}_\nu \right\rangle}{\sum\limits_{\nu,\mu=1}^{N} \Gamma_\nu \Gamma_\mu \left\langle \hat{\mathcal{O}}_\nu^\dagger \hat{\mathcal{O}}_\mu^\dagger \hat{\mathcal{O}}_\mu \hat{\mathcal{O}}_\nu \right\rangle} \tag{1}$$

on the fully excited state. This is shown in Supp. Figure 2(a) at the critical distance for different arrays. We note that the Hamiltonian causes very slow dephasing in the case of a linear or square array, and has no impact on the ring array. Calculations show that mixing due to non-measurement introduces an additional (but smaller) dephasing.

The dephasing is reduced with N, as shown in Supplementary Figure 2(b) at the critical distance. Hamiltonian dephasing is primarily due to inhomogeneous (i.e., local) frequency shifts caused by interactions [5]. With increasing N, d_{critical} increases, such that interactions are reduced at the critical distance and dephasing is reduced. Furthermore, atoms that see the similar local environment have similar shifts. This means that the inhomogeneity reduces as N increases, as the fraction of atoms in the "bulk" vs the edges increases with N. This effect is more pronounced for the chain, where the fraction of bulk atoms scales as $1/N$, than the square array, where the fraction scales as $1/\sqrt{N}$, as can be seen in the inset to Supplementary Figure 2(c).

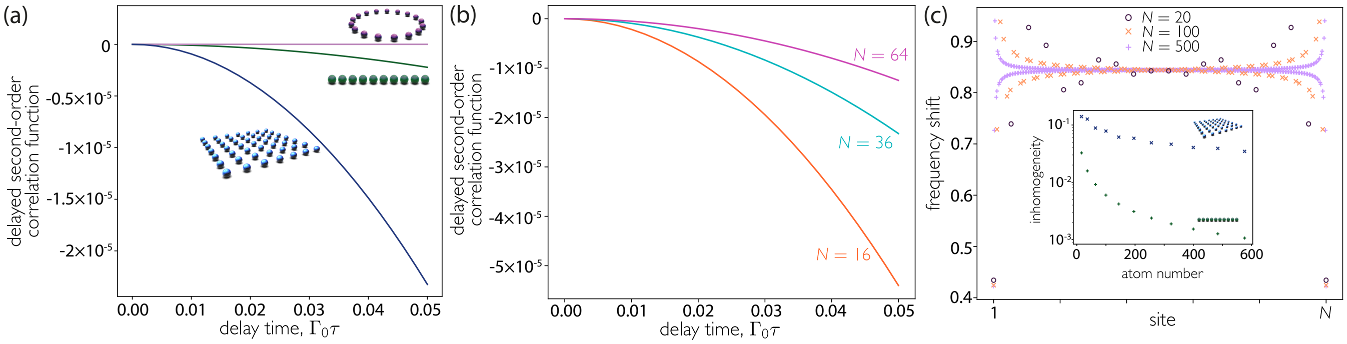

Supplementary Figure 2. Impact of Hamiltonian interactions on the second order correlation function. (a,b) $g^{(2)}(\tau)/g^{(2)}(0)-1$ is plotted as a function of delay time, showing that the Hamiltonian strictly causes dephasing, or, in the case of the ring, does not have any impact. In (a), calculations are made for different shaped arrays of 36 atoms. In (b), calculations are made for a square array of different number of atoms. In all cases, calculations are made at critical distance and the polarization axis is perpendicular to the array. (c) Frequency shifts for each atom in a linear chain. Inset shows the scaling with atom number of the variance of the frequency shifts normalized by the mean. In both plots, $d/\lambda_0 = 0.25$.

III. DERIVATION OF $g^{(2)}(0)$ FOR AN IMPERFECTLY PREPARED INITIAL STATE

Here we consider the role of "single-hole" imperfections, i.e., where not all atoms are in the excited state. This state reads

$$|\psi\rangle = \sqrt{1-\sum_{a=1}^{N}|\zeta_a|^2}\bigotimes_{n=1}^{N}|e\rangle_n + \sum_{a=1}^{N}\zeta_a\,|g\rangle_a \bigotimes_{n=1\neq a}^{N}|e\rangle_n\,, \tag{2}$$

where ζ_a is the complex coefficient for the single-hole state in which atom a is in the ground state.

The quantities required to calculate $g^{(2)}(0)$ do not mix states with different excitation numbers so we can evaluate the single-hole contribution separately to the fully-excited contribution. On the single-hole state, the expectation values required to calculate $g^{(2)}(0)$ are calculated as

$$\left(\sum_{a=1}^{N}\zeta_a^*\,\langle g|_a\bigotimes_{n\neq a}\langle e|_n\right)\hat{\sigma}_{eg}^{i}\hat{\sigma}_{ge}^{j}\left(\sum_{b=1}^{N}\zeta_b\,|g\rangle_b\bigotimes_{p\neq b}|e\rangle_p\right) = \left(\delta_{ij}\delta_{ab}+\delta_{ib}\delta_{ja}\right)\left(1-\delta_{ia}\right) \tag{3a}$$

$$\left(\sum_{a=1}^{N}\zeta_a^*\,\langle g|_a\bigotimes_{n\neq a}\langle e|_n\right)\hat{\sigma}_{eg}^{i}\hat{\sigma}_{eg}^{j}\hat{\sigma}_{ge}^{l}\hat{\sigma}_{ge}^{m}\left(\sum_{b=1}^{N}\zeta_b\,|g\rangle_b\bigotimes_{p\neq b}|e\rangle_p\right)$$
$$= \left[\delta_{ab}\left(\delta_{il}\delta_{jm}+\delta_{im}\delta_{jl}\right)+\delta_{ib}\left(\delta_{jl}\delta_{ma}+\delta_{jm}\delta_{la}\right)+\delta_{jb}\left(\delta_{il}\delta_{ma}+\delta_{im}\delta_{la}\right)\right]\left(1-\delta_{ij}\right)\left(1-\delta_{ia}\right)\left(1-\delta_{ja}\right) \tag{3b}$$

We calculate the numerator and denominator of $g^{(2)}(0)$ separately. The denominator is as follows

$$\begin{aligned}
\sum_{\nu=1}^{N}\Gamma_\nu\left\langle\hat{\mathcal{O}}_\nu^\dagger\hat{\mathcal{O}}_\nu\right\rangle &= \sum_{\nu=1}^{N}\Gamma_\nu\sum_{a,b,i,j=1}^{N}\zeta_a^*\zeta_b\alpha_{\nu,i}^*\alpha_{\nu,j}\left(\delta_{ij}\delta_{ab}+\delta_{ib}\delta_{ja}\right)\left(1-\delta_{ia}\right)\\
&= \sum_{\nu=1}^{N}\Gamma_\nu\left[\sum_{a,i=1}^{N}|\zeta_a|^2|\alpha_{\nu,i}|^2+\zeta_a^*\zeta_i\alpha_{\nu,i}^*\alpha_{\nu,a}-2\sum_{i=1}^{N}|\zeta_i|^2|\alpha_{\nu,i}|^2\right]\\
&= (N-2)\Gamma_0\sum_{a=1}^{N}|\zeta_a|^2+\sum_{a,i,\nu=1}^{N}\Gamma_\nu\zeta_a^*\zeta_i\alpha_{\nu,i}^*\alpha_{\nu,a}.
\end{aligned}\tag{4}$$

Following a similar procedure, the numerator is readily found to be

$$\begin{aligned}
\sum_{\nu,\mu=1}^{N}\Gamma_\nu\Gamma_\mu\left\langle\hat{\mathcal{O}}_\nu^\dagger\hat{\mathcal{O}}_\mu^\dagger\hat{\mathcal{O}}_\mu\hat{\mathcal{O}}_\nu\right\rangle &= \sum_{\nu,\mu=1}^{N}\Gamma_\nu\Gamma_\mu\sum_{a,b,i,j,l,m=1}^{N}\zeta_a^*\zeta_b\alpha_{\mu,i}^*\alpha_{\nu,j}^*\alpha_{\nu,l}\alpha_{\mu,m}[\delta_{ab}\left(\delta_{il}\delta_{jm}+\delta_{im}\delta_{jl}\right)+\delta_{ib}\left(\delta_{jl}\delta_{ma}+\delta_{jm}\delta_{la}\right)\\
&\quad+\delta_{jb}\left(\delta_{il}\delta_{ma}+\delta_{im}\delta_{la}\right)]\left(1-\delta_{ij}-\delta_{ia}-\delta_{ja}+2\delta_{ij}\delta_{ia}\right)\\
&= \sum_{a=1}^{N}|\zeta_a|^2\left[\left(N^2-6N+12\right)\Gamma_0^2+\sum_{\nu=1}^{N}\Gamma_\nu^2\left(1-4|\alpha_{\nu,a}|^2\right)\right]+\sum_{a,i,\nu=1}^{N}\zeta_a^*\zeta_i\left[\left(2N-8\right)\Gamma_0\Gamma_\nu+2\Gamma_\nu^2\right]\alpha_{\nu,i}^*\alpha_{\nu,a}
\end{aligned}\tag{5}$$

We can now combine these with the fully-excited results to find $g^{(2)}(0)$ for the state given by Eq. (2)

$$g^{(2)}(0)=\frac{\left(N^2-2N\right)\Gamma_0^2+\sum_{\nu=1}^{N}\Gamma_\nu^2-4\sum_{a=1}^{N}|\zeta_a|^2\left[\left(N-3\right)\Gamma_0^2+\sum_{\nu=1}^{N}\Gamma_\nu^2|\alpha_{\nu,a}|^2\right]+\sum_{a,i=1}^{N}\zeta_a^*\zeta_i\left[\sum_{\nu=1}^{N}\left(\left(2N-8\right)\Gamma_0\Gamma_\nu+2\Gamma_\nu^2\right)\alpha_{\nu,i}^*\alpha_{\nu,a}\right]}{\left[\left(N-2\sum_{a=1}^{N}|\zeta_a|^2\right)\Gamma_0+\sum_{a,i,\nu=1}^{N}\Gamma_\nu\zeta_a^*\zeta_i\alpha_{\nu,i}^*\alpha_{\nu,a}\right]^2}.\tag{6}$$

To investigate the impact of the imperfect initial state, we consider coherent spin states of the form

$$|\varphi,\mathbf{k}\rangle=\bigotimes_{j=1}^{N}\left(\sqrt{1-\varphi}\,|g\rangle_j+\mathrm{e}^{i\mathbf{k}\cdot\mathbf{r}_j}\sqrt{\varphi}\,|e\rangle_j\right).\tag{7}$$

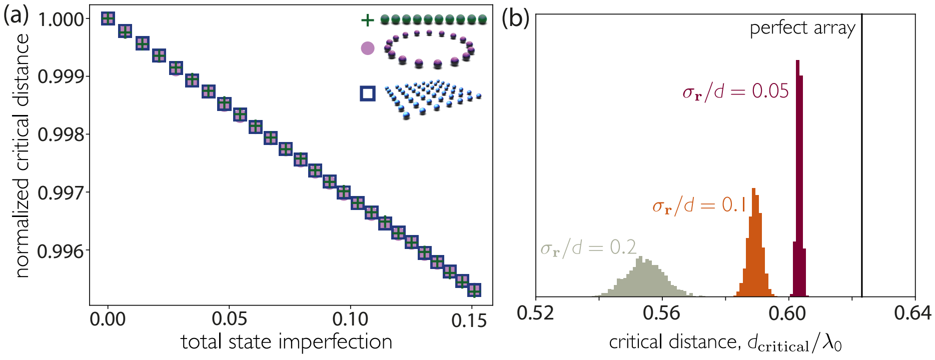

Supplementary Figure 3. (a) Impact of imperfections in the initial state on the critical distance at which superradiance disappears. The critical distance is found for different shape arrays of 36 atoms prepared in coherent spin states of the form given by Eq. (8), and plotted normalized by the critical distance for the fully inverted array. Atoms are arranged in the $x-y$ plane, or along the x-axis for the chain, with polarization axis along z and drive along x. (b) Impact of classical spatial disorder on superradiance. The histogram shows the critical distance for 2000 configurations of a 12×12 atom array with 3D Gaussian noise added to positions. Noise is added proportionally to the inter-atomic distance. Atoms are polarized perpendicular to the plane.

These would be produced experimentally by a short, intense pulse of duration $\tau \ll \{(N\Gamma_0)^{-1}, J_{12}^{-1}\}$. Here, we consider $\varphi \approx 1$ such that we truncate the state to the form (here left unnormalized for simplicity)

$$|\varphi, \mathbf{k}\rangle \approx \sqrt{\varphi^N} \bigotimes_{j=1}^{N} |e\rangle_j + \sum_{j=1}^{N} \mathrm{e}^{-i\mathbf{k}\cdot\mathbf{r}_j} \sqrt{\varphi^{N-1}(1-\varphi)}\, |g\rangle_j \bigotimes_{l\neq j} |e\rangle_l \tag{8}$$

and use Eq. (6) to calculate the critical distance for imperfect initial states. Supplementary Figure 3(a) shows that the impact is marginal. For a total imperfection of 15%, the critical distance drops by a factor of only 0.4%. For these small imperfections in the initial state, the relative decrease in d_{critical} is approximately linear, and seems to be independent of the array geometry.

IV. CRITICAL DISTANCE IN THE PRESENCE OF CLASSICAL SPATIAL DISORDER

Supplementary Figure 3(b) shows that superradiance is robust to classical disorder the position of in the emitters. We add a randomly-generated 3D Gaussian noise to each emitter position with standard deviation $\sigma_{\mathbf{r}}$ in all directions. We stochastically generate a large number of arrays and find the critical distance at which superradiance is lost.

V. CONSIDERATIONS FOR SOLID-STATE EMITTERS

Solid-state emitters constitute an alternative platform for producing emitter arrays, as strongly sub-wavelength distances can be achieved simply through fabrication, without the need for optical trapping. However, these emitters have other issues that may negatively impact collective decay. Here, we consider the impact of inhomogeneous broadening and non-radiative decay.

A. Inhomogeneous broadening

For non-identical emitters, we define each emitter to have frequency ω_0^i and spontaneous emission rate Γ_0^i, with mean values $\bar{\omega}_0$ and $\bar{\Gamma}_0$. If the frequency broadening is small, such that the spectral response is flat across the range of ω_0^i, then frequency broadening does not impact the treatment of the dissipation and we can follow the derivation of $g^{(2)}(0)$ above with the alterations that the operator decay rates now obey

$$\sum_{\nu=1}^{N} \Gamma_\nu |\alpha_{\nu,i}|^2 = \Gamma_0^i \quad \text{and} \quad \sum_{\nu=1}^{N} \Gamma_\nu = N\bar{\Gamma}_0. \tag{9}$$

Therefore

$$g^{(2)}(0) = \frac{\sum\limits_{\nu,\mu=1}^{N} \Gamma_\nu \Gamma_\mu \left(1 + \delta_{\nu\mu} - 2\sum\limits_{i=1}^{N} |\alpha_{\nu,i}|^2 |\alpha_{\mu,i}|^2\right)}{N^2 \bar{\Gamma}_0^2} = 1 + \sum_{\nu=1}^{N} \left(\frac{\Gamma_\nu}{N\bar{\Gamma}_0}\right)^2 - 2\sum_{i=1}^{N} \left(\frac{\Gamma_0^i}{N\bar{\Gamma}_0}\right)^2. \tag{10}$$

This can be recast in terms of two variances as

$$g^{(2)}(0) = 1 + \frac{1}{N}\left[\mathrm{Var}\left(\frac{\Gamma_\nu}{\bar{\Gamma}_0}\right) - 1\right] - \frac{2}{N}\mathrm{Var}\left(\frac{\Gamma_0^i}{\bar{\Gamma}_0}\right). \tag{11}$$

This expression is maximized for zero inhomogeneity, i.e. $\Gamma_0^i = \Gamma_0$, and so inhomogeneous broadening in the emitter decay rates strictly increases dephasing.

B. Non-radiative decay

Solid-state emitters can decay without emitting light. We consider that this type of decay is not correlated (i.e., it is local). The master equation thus reads

$$\dot{\rho} = -\frac{\mathrm{i}}{\hbar}[\mathscr{H}, \rho] + \sum_{\nu=1}^{N} \frac{\Gamma_\nu}{2}\left(2\hat{\mathcal{O}}_\nu \rho \hat{\mathcal{O}}_\nu^\dagger - \rho \hat{\mathcal{O}}_\nu^\dagger \hat{\mathcal{O}}_\nu - \hat{\mathcal{O}}_\nu^\dagger \hat{\mathcal{O}}_\nu \rho\right) + \sum_{i=1}^{N} \frac{\gamma_i}{2}\left(2\hat{\sigma}_{ge}^i \rho\, \hat{\sigma}_{eg}^i - \rho\, \hat{\sigma}_{eg}^i \hat{\sigma}_{ge}^i - \hat{\sigma}_{eg}^i \hat{\sigma}_{ge}^i \rho\right), \tag{12}$$

where γ_i is the non-radiative decay rate of atom i. We then write $g^{(2)}(0)$ as

$$g^{(2)}(0) = \frac{p(0,2)\sum\limits_{\nu,\mu=1}^{N}\Gamma_\nu\Gamma_\mu\langle\hat{\mathcal{O}}_\nu^\dagger\hat{\mathcal{O}}_\mu^\dagger\hat{\mathcal{O}}_\mu\hat{\mathcal{O}}_\nu\rangle + \sum\limits_{i,\nu,\mu=1}^{N}\left[p_i(1,2)\Gamma_\nu\Gamma_\mu\langle\hat{\sigma}_{eg}^i\hat{\mathcal{O}}_\nu^\dagger\hat{\mathcal{O}}_\mu^\dagger\hat{\mathcal{O}}_\mu\hat{\mathcal{O}}_\nu\hat{\sigma}_{ge}^i\rangle + p_i(2,2)\Gamma_\nu\Gamma_\mu\langle\hat{\mathcal{O}}_\nu^\dagger\hat{\sigma}_{eg}^i\hat{\mathcal{O}}_\mu^\dagger\hat{\mathcal{O}}_\mu\hat{\sigma}_{ge}^i\hat{\mathcal{O}}_\nu\rangle\right]}{\left(p(0,1)\sum\limits_{\nu=1}^{N}\Gamma_\nu\langle\hat{\mathcal{O}}_\nu^\dagger\hat{\mathcal{O}}_\nu\rangle + \sum\limits_{i,\nu=1}^{N}p_i(1,1)\Gamma_\nu\langle\hat{\sigma}_{eg}^i\hat{\mathcal{O}}_\nu^\dagger\hat{\mathcal{O}}_\nu\hat{\sigma}_{ge}^i\rangle\right)^2}, \tag{13}$$

where $p(0,j)$ is the probability of zero non-radiative events before the emission of j photons, and $p_i(l,m)$ is the probability of a single non-radiative event occurring on atom i right before the mth photon during the emission of l photons. Terms with two or more non-radiative events are assumed to be negligible and are hence ignored, as we assume the non-radiative decay to be small, $\gamma_i \ll \Gamma_0$.

We wish to expand $g^{(2)}(0)$ in the same manner as above, which requires the evaluation of the expectation values

$$\langle\hat{\sigma}_{eg}^i\hat{\sigma}_{eg}^j\hat{\sigma}_{ge}^l\hat{\sigma}_{ge}^i\rangle = \delta_{jl}\left(1-\delta_{ij}\right), \tag{14a}$$

$$\langle\hat{\sigma}_{eg}^i\hat{\sigma}_{eg}^j\hat{\sigma}_{eg}^l\hat{\sigma}_{ge}^m\hat{\sigma}_{ge}^n\hat{\sigma}_{ge}^i\rangle = \left(\delta_{jm}\delta_{ln}+\delta_{jn}\delta_{lm}\right)\left(1-\delta_{jl}\right)\left(1-\delta_{ij}\right)\left(1-\delta_{il}\right), \tag{14b}$$

$$\langle\hat{\sigma}_{eg}^j\hat{\sigma}_{eg}^i\hat{\sigma}_{eg}^l\hat{\sigma}_{ge}^m\hat{\sigma}_{ge}^i\hat{\sigma}_{ge}^n\rangle = \left(\delta_{jm}\delta_{ln}+\delta_{jn}\delta_{lm}\right)\left(1-\delta_{jl}\right)\left(1-\delta_{ij}\right)\left(1-\delta_{il}\right). \tag{14c}$$

By noting that Eqs. (14b) and (14c) yield the same result, and substituting in the expressions for terms without non-radiative terms from above, we arrive to

$$g^{(2)}(0) = \frac{p(0,2)\left(N^2\bar{\Gamma}_0^2 + \sum\limits_{\nu=1}^{N}\Gamma_\nu^2 - 2\sum\limits_{i=1}^{N}\left(\Gamma_0^i\right)^2\right) + \sum\limits_{i,\nu,\mu=1}^{N}\left[p_i(1,2)+p_i(2,2)\right]\Gamma_\nu\Gamma_\mu\langle\hat{\sigma}_{eg}^i\hat{\mathcal{O}}_\nu^\dagger\hat{\mathcal{O}}_\mu^\dagger\hat{\mathcal{O}}_\mu\hat{\mathcal{O}}_\nu\hat{\sigma}_{ge}^i\rangle}{\left(p(0,1)N\Gamma_0 + \sum\limits_{i,\nu=1}^{N}p_i(1,1)\Gamma_\nu\langle\hat{\sigma}_{eg}^i\hat{\mathcal{O}}_\nu^\dagger\hat{\mathcal{O}}_\nu\hat{\sigma}_{ge}^i\rangle\right)^2}. \tag{15}$$

We are interested in calculating $g^{(2)}(0)$ around the critical distance, where the second photon is emitted at approximately the same rate as the first, $N\Gamma_0$. In this situation, we can approximate the probabilities as

$$p(0,1) = \frac{N\bar{\Gamma}_0}{N\bar{\Gamma}_0 + N\bar{\gamma}}, \tag{16a}$$

$$p_i(1,1) = \frac{\gamma_i}{N\bar{\Gamma}_0 + N\bar{\gamma}}, \tag{16b}$$

$$p(0,2) = \frac{N\bar{\Gamma}_0}{N\bar{\Gamma}_0 + 2N\bar{\gamma}}, \tag{16c}$$

$$p_i(1,2) = \frac{\gamma_i}{N\bar{\Gamma}_0 + 2N\bar{\gamma}}, \tag{16d}$$

$$p_i(2,2) = \frac{\gamma_i}{N\bar{\Gamma}_0 + 2N\bar{\gamma}} = p_i(1,2), \tag{16e}$$

where $\bar{\gamma}$ is the mean non-radiative decay rate. This approximation should also be valid for large N, where the emission of the first photon does not substantially alter the rate of the second photon. This simplifies the expression to

$$g^{(2)}(0) = \frac{p(0,2)\left(N^2\bar{\Gamma}_0^2 + \sum\limits_{\nu=1}^{N}\Gamma_\nu^2 - 2\sum\limits_{i=1}^{N}\left(\Gamma_0^i\right)^2\right) + 2\sum\limits_{i,\nu,\mu=1}^{N}p_i(1,2)\Gamma_\nu\Gamma_\mu\langle\hat{\sigma}_{eg}^i\hat{\mathcal{O}}_\nu^\dagger\hat{\mathcal{O}}_\mu^\dagger\hat{\mathcal{O}}_\mu\hat{\mathcal{O}}_\nu\hat{\sigma}_{ge}^i\rangle}{\left(p(0,1)N\Gamma_0 + \sum\limits_{i,\nu=1}^{N}p_i(1,1)\Gamma_\nu\langle\hat{\sigma}_{eg}^i\hat{\mathcal{O}}_\nu^\dagger\hat{\mathcal{O}}_\nu\hat{\sigma}_{ge}^i\rangle\right)^2}. \tag{17}$$

We thus need to calculate

$$\sum_{i,\nu=1}^{N}p_i(1,1)\Gamma_\nu\langle\hat{\sigma}_{eg}^i\hat{\mathcal{O}}_\nu^\dagger\hat{\mathcal{O}}_\nu\hat{\sigma}_{ge}^i\rangle = \sum_{i,j,l,\nu=1}^{N}p_i(1,1)\Gamma_\nu\alpha_{\nu,j}^*\alpha_{\nu,l}\langle\hat{\sigma}_{eg}^i\hat{\sigma}_{eg}^j\hat{\sigma}_{ge}^l\hat{\sigma}_{ge}^i\rangle = \sum_{i,j,l,\nu=1}^{N}p_i(1,1)\Gamma_\nu\alpha_{\nu,j}^*\alpha_{\nu,l}\delta_{jl}\left(1-\delta_{ij}\right)$$

$$= \sum_{i,j,\nu=1}^{N}p_i(1,1)\Gamma_\nu|\alpha_{\nu,j}|^2 - \sum_{i,\nu=1}^{N}p_i(1,1)\Gamma_\nu|\alpha_{\nu,i}|^2 = (N-1)\sum_{i=1}^{N}p_i(1,1)\Gamma_0^i, \tag{18}$$

and

$$\sum_{i,\nu,\mu=1}^{N} p_i(1,2)\Gamma_\nu\Gamma_\mu \langle \hat{\sigma}^i_{eg}\hat{\mathcal{G}}^\dagger_\nu\hat{\mathcal{G}}^\dagger_\mu\hat{\mathcal{G}}_\mu\hat{\mathcal{G}}_\nu\hat{\sigma}^i_{ge}\rangle = \sum_{i,\nu,\mu=1}^{N} p_i(1,2)\Gamma_\nu\Gamma_\mu\alpha^*_{\nu,j}\alpha^*_{\mu,l}\alpha_{\mu,m}\alpha_{\nu,n} \langle \hat{\sigma}^i_{eg}\hat{\sigma}^j_{eg}\hat{\sigma}^l_{eg}\hat{\sigma}^m_{ge}\hat{\sigma}^n_{ge}\hat{\sigma}^i_{ge}\rangle$$

$$= \sum_{i,\nu,\mu=1}^{N} p_i(1,2)\Gamma_\nu\Gamma_\mu\alpha^*_{\nu,j}\alpha^*_{\mu,l}\alpha_{\mu,m}\alpha_{\nu,n} \left(\delta_{jm}\delta_{ln}+\delta_{jn}\delta_{lm}\right)\left(1-\delta_{jl}\right)\left(1-\delta_{ij}\right)\left(1-\delta_{il}\right)$$

$$= \sum_{i=1}^{N} p_i(1,2)\left[N^2\bar{\Gamma}_0^2 + \sum_{\nu=1}^{N}\Gamma_\nu^2\left(1-2|\alpha_{\nu,i}|^2\right) - 2N\Gamma_0^i\bar{\Gamma}_0 + 4\left(\Gamma_0^i\right)^2 - 2\sum_{j=1}^{N}\left(\Gamma_0^j\right)^2\right]. \tag{19}$$

Combining these two expressions we obtain the second order correlation function near the critical distance as

$$g^{(2)}(0) = \tag{20}$$

$$\frac{\left(\bar{\Gamma}_0+2\bar{\gamma}\right)N^2\bar{\Gamma}_0^2 + \sum_{\nu=1}^{N}\Gamma_\nu^2\left(\bar{\Gamma}_0+2\bar{\gamma}-\frac{4}{N}\sum_{i=1}^{N}\gamma_i|\alpha_{\nu,i}|^2\right) + \sum_{i=1}^{N}\left(\frac{8\gamma_i}{N}-2\bar{\Gamma}_0\right)\left(\Gamma_0^i\right)^2 - \sum_{i=1}^{N}4\gamma_i\left(\Gamma_0^i\bar{\Gamma}_0+\sum_{j=1}^{N}\frac{\left(\Gamma_0^j\right)^2}{N}\right)}{\frac{\bar{\Gamma}_0+2\bar{\gamma}}{\left(N\bar{\Gamma}_0+N\bar{\gamma}\right)^2}\left[N^2\bar{\Gamma}_0^2+(N-1)\sum_{i=1}^{N}\gamma_i\Gamma_0^i\right]^2}.$$

If each emitter has the same non-radiative decay rate γ, this simplifies to

$$g^{(2)}(0) = \left(1+\frac{\gamma}{\bar{\Gamma}_0}\right)^2\left(1-\frac{4\gamma}{N\bar{\Gamma}_0+2N\gamma}\right)\frac{N^2\bar{\Gamma}_0^2+\sum_{\nu=1}^{N}\Gamma_\nu^2-2\sum_{i=1}^{N}\left(\Gamma_0^i\right)^2}{\left[N\bar{\Gamma}_0+(N-1)\gamma\right]^2}. \tag{21}$$

C. Superradiance with solid-state emitters

Superradiance persists in the presence of non-radiative decay and inhomogeneous broadening. Supplementary Figure 4(a) shows that the superradiant burst survives levels of non-radiative decay as large as those of radiative decay. Nevertheless, increased non-radiative decay rates enhance dephasing, eventually destroying superradiance as the emission pathways are dominated by non-radiative routes. As a result, the critical distance at which the superradiant burst disappears is shifted to smaller distances. Supplementary Figure 4(b) shows that the superradiant burst survives inhomogeneous broadening on the emitter resonance frequency even at levels beyond 10 times the

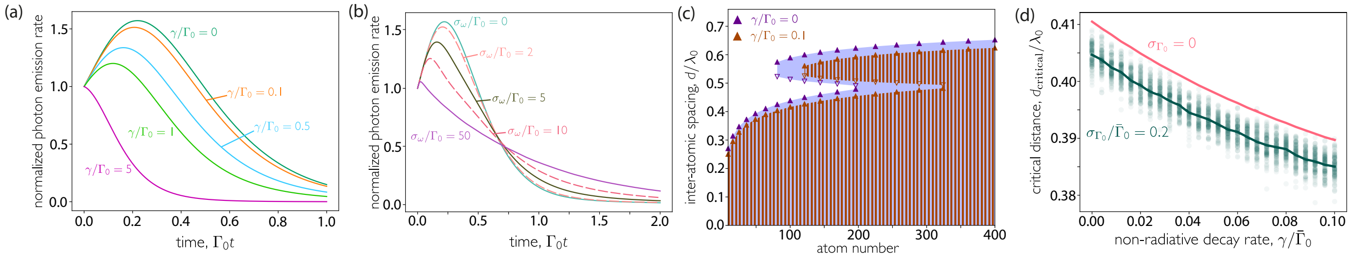

Supplementary Figure 4. Impact of non-radiative decay and inhomogeneous broadening on superradiance. (a,b) Photon emission rate from an initially inverted square array of 3×3 emitters and inter-atomic spacing $d = 0.1\lambda_0$ in the presence of (a) non-radiative decay and (b) inhomogeneous broadening on the emitter resonance frequencies. In (b), plotted curves are the average of 100 stochastically generated instances with Gaussian distributed noise of width σ_ω. (c) Boundaries between the burst (colored) and no-burst (white) regions as a function of inter-particle distance d and emitter number for square arrays with and without non-radiative decay. The symbols $\triangle$ and $\bigtriangledown$ represent points where, with decreasing d, $g^{(2)}(0)$ goes above and below unity, respectively. (d) Critical distance for square arrays of 8×8 emitters as a function of non-radiative decay rate. In the presence of inhomogeneous broadening, the decay rate of each emitter is calculated as a random sample of a Gaussian distribution with mean Γ_0 and standard deviation σ_{Γ_0}. Circles represent individual stochastic samples, and the solid line shows the average of 100 samples. In all cases, emitters are polarized perpendicular to the array and are assumed to have the same non-radiative decay rate γ.

linewidth. The burst is diminished in size and duration, but not destroyed. Supplementary Figure 4(c) shows that non-radiative decay always provides a stricter bound on superradiance, although the impact is relatively small if radiative decay is still the dominant decay mechanism. As the level of non-radiative decay increases, the critical distance decreases, as shown in Supplementary Figure 4(d). The addition of inhomogeneous broadening on the atoms' linewidths results in a further small decrease in the critical distance.

SUPPLEMENTARY REFERENCES

[1] Bettles, R. J., Gardiner, S. A. & Adams, C. S. Cooperative ordering in lattices of interacting two-level dipoles. *Phys. Rev. A* **92**, 063822 (2015)..

[2] Bettles, R. J., Gardiner, S. A. & Adams, C. S. Enhanced optical cross section via collective coupling of atomic dipoles in a 2D array. *Phys. Rev. Lett.* **116**, 103602 (2016).

[3] Krämer, S., Ostermann, L. & Ritsch, H. Optimized geometries for future generation optical lattice clocks. *EPL (Europhysics Letters)* **114**, 14003 (2016).

[4] Javanainen, J. & Rajapakse, R. Light propagation in systems involving two-dimensional atomic lattices. *Phys. Rev. A* **100**, 013616 (2019).

[5] Friedberg, R., Hartmann, S. R. & Manassah, J. T. Limited superradiant damping of small samples. *Phys. Lett. A* **40**, 365–366 (1972).
